# Challenges and constraints to the sustainability of poultry farming in Indonesia

**DOI:** 10.5713/ab.24.0678

**Published:** 2025-02-25

**Authors:** Sumiati, Roni Fadilah, Arif Darmawan, Rizky Nadia

**Affiliations:** 1 Department of Nutrition and Feed Technology, Faculty of Animal Science, IPB University, Bogor, Indonesia; 2 New Hope Indonesia, Tangerang Regency, Indonesia

**Keywords:** Biosecurity, Challenges, Indonesia, Poultry Population, Poultry Production, Solutions

## Abstract

Although the demand for poultry products in Indonesia, one of the most significant poultry-producing countries in Southeast Asia, continues to increase, the poultry farming sector continues to face various challenges. This paper provides an overview of the prospects for and challenges faced by the poultry farming sector in Indonesia. Broilers comprise the largest portion of Indonesia’s poultry population, with other commodities contributing less than 10% of the total. During the COVID-19 pandemic, the consumption of chicken meat and eggs increased. The declining national economy led people to consume affordable food for their daily meals, favoring chicken and eggs because their retail prices were four times cheaper than that of beef. In terms of imports to Indonesia, compared with other agricultural commodities, poultry and eggs account for only approximately 1.26% and 0.41%, respectively. Meanwhile, some feed ingredients, such as soybean meal and corn, continue to be imported into Indonesia. According to data from the United States Department of Agriculture, Indonesia imported 4.4 million tons of soybean meal to meet the needs of its poultry industry. In recent years, feed mill companies have rapidly been developing in Indonesia, with an estimated average production of 572,000 metric tons. In terms of scale, poultry farming in Indonesia comprises 60% large-scale farming and 40% small- and medium-scale farming. Many small-scale farms have yet to implement strict biosecurity measures, making climate change a significant challenge for poultry farming in Indonesia. After implementing the ban on antibiotic growth promoters, Indonesia’s poultry farming sector began to stabilize and look for alternative methods to maintain productivity. The use of technology, improved regulations, and the enhancement of farmers’ capacity to manage challenges are key toward sustaining and increasing poultry farming productivity in Indonesia in the future.

## INTRODUCTION

Poultry farming is a crucial sector of the Indonesian economy, particularly as a source of animal protein. Chicken meat and eggs are Indonesia’s most consumed poultry products, primarily due to their relatively affordable prices compared with other protein sources, such as beef [1]. As Indonesia’s population grows and its purchasing power increases, demand for chicken meat and eggs continues to rise [2]. However, various challenges could hinder the sustainability of Indonesia’s poultry farming sector.

One challenge facing the industry is the fluctuating price of feed, the largest component of poultry production costs. Most feedstuffs, such as corn and soybean meal (SBM), continue to be imported in Indonesia, making feed prices highly dependent on international market dynamics [3,4]. This dependency results in considerable fluctuations in feed prices, significantly affecting production costs and market prices. Meanwhile, climate change represents a significant external challenge to the sustainability of poultry farming in Indonesia [5]. Higher temperatures due to global warming and unpredictable weather patterns can result in heat stress, which can reduce productivity, lessen growth, and increase mortality rates [6]. This impact is more pronounced for smallholder farmers who cannot afford climate control technology. In terms of regulations, the government’s ban on antibiotic growth promoters (AGPs) has created new challenges for farmers. Although this policy aims to reduce the risk of antibiotic resistance in humans, many farmers have faced difficulty in maintaining poultry health without using AGPs [7]. Therefore, many farmers have sought alternatives such as probiotics and natural feed additives; however, the effectiveness of these alternatives in maintaining productivity is not always optimal and often constrained by high costs and availability [8]. The situation is further exacerbated by the low implementation of biosecurity measures at some farms, especially small-scale farms, which increases the risk of disease spread [9]. This paper presents a brief overview of the poultry industry in Indonesia, including future prospects, opportunities, challenges, and solutions to support the sustainability of the poultry industry in Indonesia.

## CURRENT SITUATIONS

### Poultry population in Indonesia

Indonesia’s poultry industry plays a vital role in the country’s agricultural sector, supplying crucial protein to millions of people through products such as chicken meat and eggs. In recent years, the industry has experienced significant growth, driven by rising domestic demand due to population growth, urbanization, and increased incomes [10]. Indonesia is one of the largest poultry producers in Southeast Asia, featuring a diverse array of farms, from large-scale commercial operations to small backyard setups.

**Figure 1:**
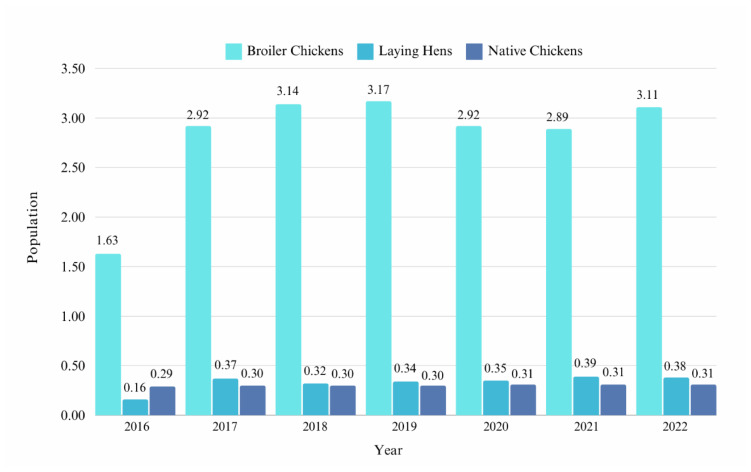
Population of broiler chickens, laying hens, and native chicken (billion heads) in Indonesia (2016–2022). Data from Direktorat Statistik Peternakan, Perikanan, dan Kehutanan [2].

**Figure 2:**
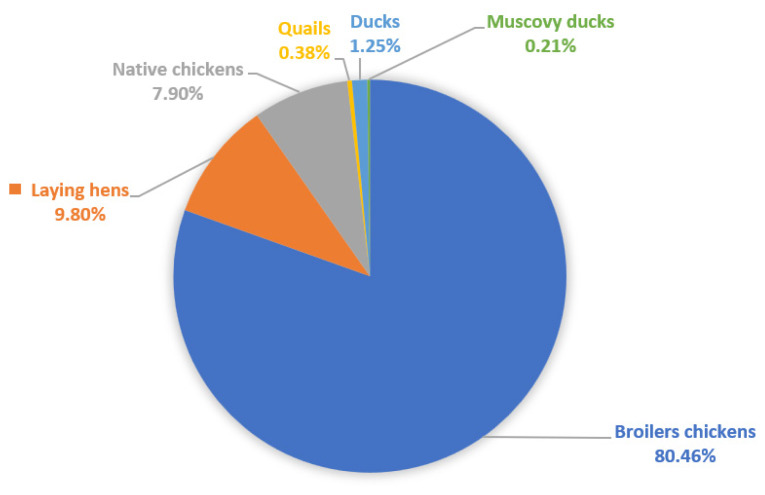
Percentage of poultry population by type in 2022. Data from Direktorat Statistik Peternakan, Perikanan, dan Kehutanan [2].

In 2022, Indonesia had a population of 3.11 billion broiler chickens, 0.37 billion laying hens, 0.3 billion native chickens, and 56 million ducks (including Muscovy ducks). The broiler population has increased nearly every year, except for a decrease of 7.8% from 2019 to 2020. Figure 1 presents the populations of broiler chickens, laying hens, and native chickens in Indonesia. Broilers account for the largest share of the poultry population at 80%. Several broiler chicken strains can be found in Indonesia, including Cobb, Ross, Indian River, Hubbard [11], Lohmann, and Hybro. Meanwhile, layer and native chickens constitute less than 10% of the population, whereas other commodities, such as ducks and quails, constitute less than 2% to the total poultry population [2]. Figure 2 presents the poultry population (in percentages) by type.

### Consumption, supply, and demand of broiler chicken meat

**Figure 3:**
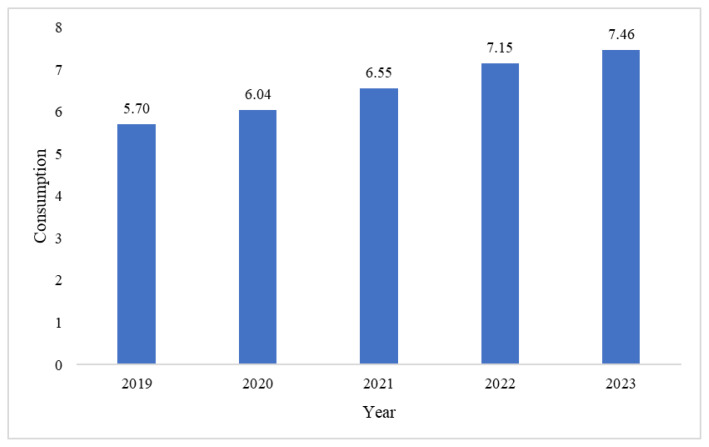
Broiler meat consumption in Indonesia (kg per capita per year), 2019–2023. Data from Ministry of Agriculture [21].

**Figure 4:**
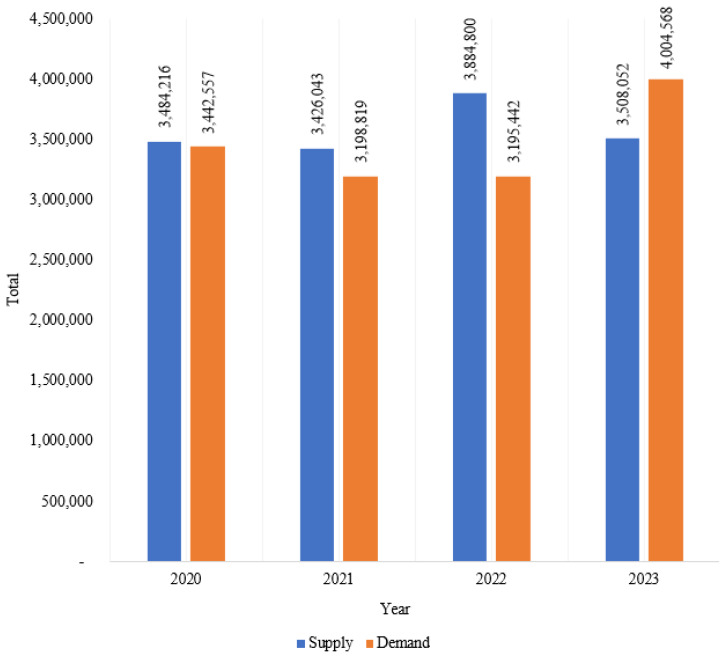
Supply and demand for chicken meat (tons) in 2020–2023. Data from Indrawan et al [9], Fukase and Martin [10], Rofii et al [11], and Subdirectorate of Livestock Statistics [12].

Poultry meat consumption, especially broiler meat, has increased from year to year in line with the country’s growing population. In 2019, broiler meat consumption was 5.7 kg per capita per year and consistently increased to 7.46 kg per capita per year in 2023 (Figure 3). However, this consumption is only household consumption, excluding stalls, restaurants, and hotels. Figure 4 illustrates the supply and demand trends for broiler chicken meat in Indonesia from 2020 to 2023. Throughout this period, the supply is generally able to meet the demand for broiler chicken meat. In 2020, the supply was almost 3.5 million tons, while demand 3.4 million tons [12]. However, in 2021, both supply and demand declined 1.66% and 7.08%, respectively [13]. By 2022, supply increased but the demand was almost same as the previous year [14]. From 2020 to 2022, broiler meats indicated a slight surplus. In 2023, the demand exceeded the supply, with values at 4 million tons and 3.5 million tons, respectively [2].

In addition to the needs of the community, several factors affect the availability of broiler chicken meat:

a. Domestic production: chicken production in Indonesia is influenced by farm capacity, technology adoption, feed quality, and animal health conditions.
b. Technology and infrastructure: modern technologies such as closed-house systems can improve production efficiency and chicken meat quality.
c. Government policies: subsidies, technical support, and veterinary health regulations play a role in improving production.
d. Weather and climate: these factors can affect poultry production, especially in areas with open-house systems.
e. Imports: the availability of imported chicken can increase domestic supply, especially when local production is insufficient.

### Egg consumption, supply, and demand

**Figure 5:**
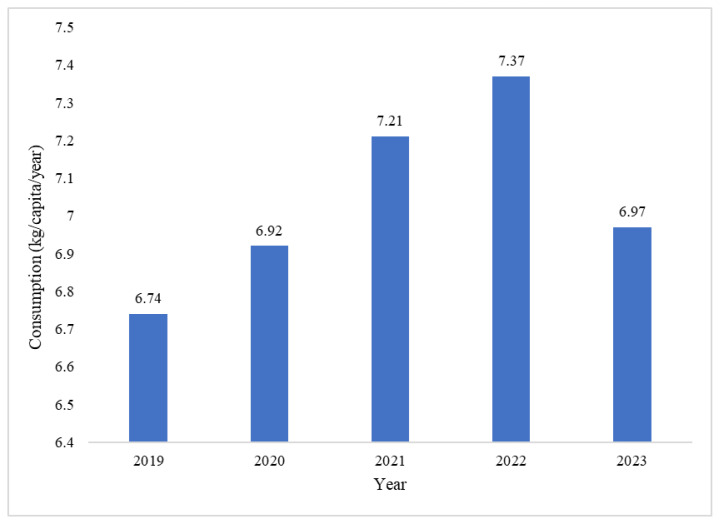
Egg consumption in Indonesia (kg per capita per year), 2019–2023. Data from Ministry of Agriculture [21].

Eggs remain the first choice of most people in fulfilling their protein needs because of egg’s affordable price. Between 2019 and 2023, egg consumption in Indonesia showed a significant upward trend, reflecting a change in consumption habits. In 2019, egg consumption was approximately 6.74 kg per capita per year (Figure 5). In the following years, this figure has continued to increase due to several factors, such as increased awareness of the nutritional value of eggs, population growth, and economic development. By 2022, egg consumption had increased to approximately 7.37 kg per capita per year, highlighting a significant shift in consumption habits over five years. In particular, the Coronavirus disease 2019 (COVID-19) pandemic greatly influenced the increase in egg consumption. In addition, efforts made by the Indonesian government and various other organizations to promote poultry farming and egg production across the country also contributed to this increase. Meanwhile, improvements in infrastructure, such as better distribution networks and the increased availability of feed, have contributed to enhance egg distribution. Campaigns highlighting the nutritional value of eggs, including their protein content and affordability, have further boosted their popularity among Indonesian consumers. As such, the rising egg consumption trend is expected to continue, supported by ongoing efforts to enhance production and distribution capabilities.

**Figure 6:**
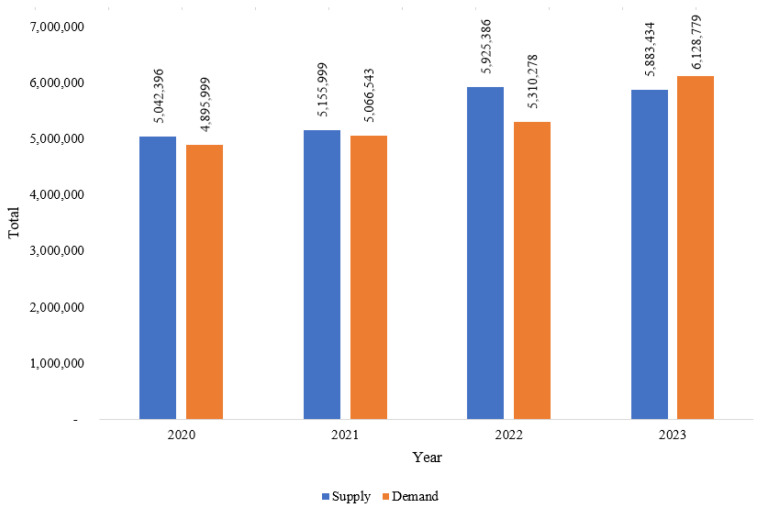
Supply and demand of laying hen eggs (tons) in 2020–2023. Data from Indrawan et al [9], Fukase and Martin [10], Rofii et al [11], and Subdirectorate of Livestock Statistics [12].

Figure 6 shows the supply and demand for laying hen eggs in Indonesia from 2020 to 2023. In this period, the supply consistently exceeded the demand. In 2020, the supply was 5 million tons, while demand reached 4.9 million tons [12]. Both supply and demand increased slightly in 2021 [13]. In 2022, there was a more significant rose in supply, for 14.9%, while demand increased for 4.81% [14]. By 2023, demand showed notable growth, surpassing 6 million ton, while supply also increased to 5.8 million tons. This trend highlights a steady rise in both the supply and demand for laying hen eggs, with demand growing at a slightly faster rate.

### Poultry commodity trading

According to the Directorate General of Animal Husbandry and Animal Health of the Ministry of Agriculture [15], Indonesia must import grandparent stock (GPS) chickens in the form of day-old chicks (DOCs) every year to meet local demand for broilers and layers. The need to import GPS chickens is based on the technical calculations in the national production plan for National Stock Replacement mandated by Minister of Agriculture Regulation No. 32/2017 [15].

In 2021–2023, GPS DOC imports fluctuated, decreasing from 649,000 birds in 2021 to 641,000 birds in 2022 before increasing to 691,000 birds in 2023 (Table 1). The number of GPS broilers was determined for each farmer in 2021, based on the decree of the Directorate General of Livestock and Animal Health on Standard Operating Procedures, including ownership and/or control of poultry slaughterhouses and cold chain facilities, the performance of GPS/PS poultry farms, and the export of poultry chicks and products.

Meanwhile, the volume of poultry imports and exports in Indonesia decreased in 2021 and then increased in 2022 (Table 2). Indonesia exported a considerable amount of chicken meat, with an increase in 2021, whereas imports of chicken meat declined from 2020 to 2022 in response to increased domestic chicken meat production. Indonesia exported a significant amount of poultry meat to several countries, including Saudi Arabia, Nigeria, Egypt, Turkey, and Croatia [16]. However, annual egg imports continue to increase to meet demand, and egg exports were expected to decline from 2020 to 2022 [2].

Indonesia’s poultry industry continues to depend on imported feed raw materials, particularly SBM and corn. The main suppliers of SBM to Indonesia are Argentina, Brazil, and the United States. According to data from the Central Bureau of Statistics [17], soybean imports to Indonesia increased compared with 2021 in 2022 and 2023. The volume of soybean imports in 2021 was 722,000 tons, which increased to 724,000 tons in 2022 and 2023 (Table 3). Moreover, according to data from the United States Department of Agriculture (USDA), Indonesia imported about 4.4 million tons of SBM in 2022. This dependency is due to insufficient domestic soybean production in Indonesia, making imports essential to meet the needs of the country’s growing poultry sector.

Indonesia has three primary corn-producing areas, namely, Java (40%), Sulawesi (24%), and Nusa Tenggara (10%) [18]. Domestic production, however, often proves insufficient to meet high demand for poultry feed. Consequently, Indonesia imports corn and other essential ingredients for poultry feed. A report from the USDA [18] indicates that Indonesia’s corn imports remain considerable, particularly from Brazil and Argentina, and complement local production, despite ongoing initiatives by the government to enhance domestic corn cultivation and reduce the reliance on imports.

## OPPORTUNITIES

Poultry farming in Indonesia has a number of opportunities driven by various socioeconomic and policy factors. With the large and growing population, demand for affordable protein sources like chicken meat and eggs is consistently high, as these products remain inexpensive compared with other protein options. Meanwhile, growth in the Indonesian poultry sector is characterized by differences in productivity between large-scale farms smallholder farms. Government policies to support the poultry sector, such as through subsidies, biosecurity implementation, and infrastructure development, offer promising prospects for growth and improved efficiency. Finally, the integration of modern technology and better supply chain management along with government intervention provides significant opportunities to increase productivity and meet growing demand in domestic and export markets.

### The growing Indonesia population

Population data can be the main reference for the government in planning and evaluating development based on assumptions about components of the population growth rate, such as births, deaths, and migration [19]. Indonesia’s rapid population growth provides excellent opportunities for the growth of poultry farming.

**Figure 7:**
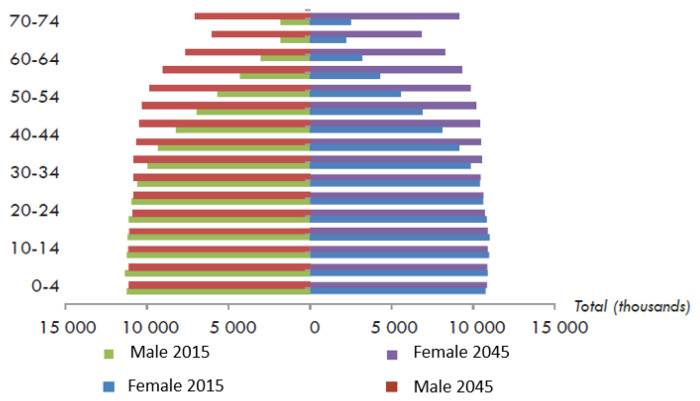
Indonesia population projections, 2015–2045. Adapted from BPS-Statistics Indonesia [19] with permission of author.

Indonesia’s population in 2024 is around 281.6 million, and it ranks fourth globally [19]. The Central Bureau of Statistics has made several Indonesia population projection to plan and make policy in developing program. The formulation of population projection 2015–2045 uses assumption with two scenarios, which are scenario A is based on assumptions relatef to policy and scenario B is based on trend [19]. Scenario A estimates a population growth of 1.00% from 2015 to 2025, whereas scenario B envisages a growth of 0.99% (Figure 7) [19]. In scenario A, Indonesia’s population in 2030 and 2045 would be 294.1 and 318.9 million people, respectively. Indonesia’s increasing population has a significant impact on poultry farming. In particular, the growing population has resulted in an increased demand for affordable and accessible protein sources, especially from chicken meat and eggs. Poultry farming is predicted to grow to meet the increased demand, potentially increasing economic growth and creating jobs in rural areas.

### The high price of non-poultry animal product

In addition to population and economic improvement, cultural preferences are positively correlated with consumption of poultry products. The cultural preference for poultry products as traditional dishes and growing awareness about their nutritional benefits have contributed to this positive trend. In Indonesia, the total consumption of protein sources such as beef, chicken, eggs, and tofu has increased significantly due to price differences and cultural preferences. Chicken meat is the most commonly consumed source of protein due to its affordability and wide availability [20]. Broiler meat, eggs, and beef per kilogram cost approximately US$2, US$1.77, and US$8, respectively [21]. As beef is priced at four times the cost of broiler meat, it is a less affordable option for daily meals (Table 4). In particular, during the COVID-19 pandemic, people were looking for affordable, nutritious options; further, the majority of the Muslim population in Indonesia preferred eggs and chicken over pork.

### Feed mills in Indonesia

Feed mill companies have rapidly developed in Indonesia, corresponding to the increased demand for poultry feed. In 2023, Indonesia had 107 feed mill factories, at both large and small scales, with an average production capacity of 702,000 metric tons (MT) and an estimated average production of 572,000 MT (Table 5) [22].

### Poultry farming scale in Indonesia

Large- and small-scale poultry farmers in Indonesia are significantly different in terms of business scale, adoption of technology, access to capital, and distribution networks [23]. Large poultry farmers have advantages in production efficiency and market penetration; meanwhile, small poultry farmers play an important role in meeting local needs and supporting the rural economy [9]. Small farms are usually managed by families or individuals with simpler management and represent small-scale operations with relatively few livestock [24]. Meanwhile, large breeders are professionally managed by staff with special skills and have a wide distribution network that can even reach foreign countries.

According to data from the Poultry Farmers Association, around 200 to 300 large poultry farmers control most poultry production in Indonesia. These farmers are typically affiliated with large integrated companies such as PT Charoen Pokphand Indonesia and PT Japfa Comfeed Indonesia, and other multinational companies. Meanwhile, based on data from the Ministry of Agriculture, the number of small poultry farmers in Indonesia is estimated to exceed 1 million. They are spread across various regions, particularly rural areas, and represent the backbone of the country’s poultry production. It is estimated that 60% of poultry production comes from industrialized farms (closed-house systems) while 40% remains in the hands of small and medium players (open-house system) [25].

### Technology

One poultry farming technology in Indonesia is the closed-house system. Housing is a crucial aspect of poultry farming. Some farms in Indonesia have started to use closed-house systems, mostly operated through corporate partnerships [26]. These collaborations reduce construction and maintenance costs. Closed-house systems offer numerous benefits, such as the ability to maintain an optimal environment by regulating heat, moisture, and harmful gases (CO, CO_2_, and NH_3_) [27]. Moreover, closed-house systems can provide better feed conversion ratios, lower mortality rate, greater profitability, and improved performance compared with open-house systems [9,23].

## CHALLENGES AND SOLUTIONS

Indonesia’s poultry farming industry faces various challenges, such as climate change, which affects the health and productivity of poultry, and poor biosecurity, increasing the risk of disease transmission. The ban on AGPs also presents an obstacle, as farmers must find alternatives to maintain their poultry’s growth and health. In addition, outbreaks of diseases such as avian influenza and Newcastle disease often lead to significant economic losses. Overcoming these obstacles requires improved strict biosecurity practices, the adoption of environmentally friendly technologies, and the use of safe and effective alternative feed materials. Efforts to increase vaccination and strict disease monitoring must also be enhanced to prevent and control disease outbreaks in poultry.

### Climate changes in Indonesia

Indonesia is a tropical country with only two seasons, the rainy and dry seasons. Extreme changes in temperature and humidity can adversely affect various sectors, especially the livestock sector. Since the industrial revolution, greenhouse gases such as carbon dioxide (CO_2_), methane (CH_4_), and nitrous oxide (N_2_O) have increased in the atmosphere and led to climate change. CH_4_ and CO_2_ levels in the air cause increased temperatures on the Earth’s surface, and the livestock sector is considered to be the largest global contributor of CH_4_ and nitrous oxide at around 10% to 12% of anthropogenic emissions [28]. Therefore, climate change greatly affects poultry production, especially in tropical countries including Indonesia.

Layer and broiler chickens are quite sensitive to the climatic environment and maintain their internal temperatures through physiological and behavioral thermoregulation. Through physiological thermoregulation, chickens alter their metabolism to control their body temperature [29]. Ambient temperatures above 30°C generally have a negative impact on chickens and lead to reduced feed consumption and body weight and increased mortality. Moreover, heat stress can also affect the quality of poultry meat, such as a significantly worsened appearance, texture, and flavor [30].

Heat stress not only reduces feed consumption and body weight but also disrupts egg production by laying hens [31]. Heat stress in laying hens can disrupt egg-producing hormones such as luteinizing hormone, reduce ovulation function, and result in poor egg quality. An increase in ambient temperature can cause hens to increase their respiratory rate to 151 times per minute [32], which can reduce the availability of blood bicarbonate for eggshell mineralization and ultimately affect egg production negatively [33].

**Figure 8:**
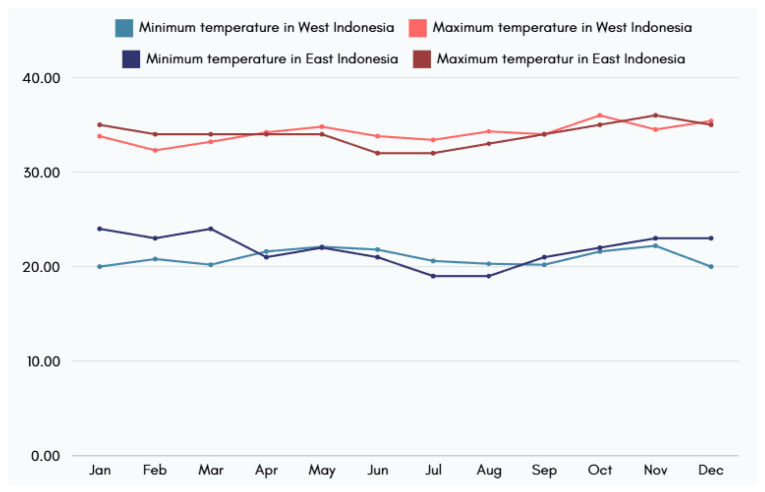
Minimum and maximum temperature (°C) in Indonesia in 2023. Data from Badan Pusat Statistik Kabupaten Pelalawan [46] and Badan Pusat Statistik Kabupaten Bogor [47].

Temperature fluctuations in Indonesia contribute to failures in optimal poultry production. For instance, the minimum temperature is around 22°C, but the maximum temperature can reach 36°C in the western and eastern parts of the country (Figure 8). The heat stress index can exceed 160 in certain months (Table 6). This condition is uncomfortable for birds and leads to decreased production. Closed- or semiclosed-house systems can be a solution to climate change in Indonesia. Alternative solutions that can be implemented by large-scale farmers are using solar energy for lighting and temperature regulation, improving waste management by processing it into fertilizer [34], and government support for sustainable practices, such as tax incentives for renewable energy adoption or subsidies for climate-resilient infrastructure.

### Poor implementation of biosecurity

Based on the how strictly they implement biosecurity measures, poultry farms in Indonesia are categorized into four sectors [35]. The modern farms in Sector 1 are run by large multinational companies that have implemented very strict biosecurity measures. Meanwhile, the commercial farms in Sector 2 have implemented medium to high biosecurity, where chickens are kept in closed areas to prevent contact with wild animals. Sector 3 comprises commercial farms, often found in Indonesia, that feature simple biosecurity measures with open-house systems that allow chickens to remain in contact with wild animals. Finally, farms in Sector 4 continue to use traditional systems without implementing biosecurity measures and are dominated by small-scale farms that often lack the resources and knowledge to implement effective protocols. These farms may not have appropriate facilities for quarantine, disinfection, or controlled access, increasing the risk of disease transmission. Furthermore, inadequate vaccination coverage and improper handling of sick or dead birds exacerbate the problem. Consequently, even farms with stringent biosecurity measures are at risk of potential contamination from neighboring operations with lower standards. According to Ilham [36], one of the causes of the weak biosecurity in Sectors 3 and 4 is limited funds, due to which farmers are unable to implement efficient and effective biosecurity. However, the government has begun to address this issue through activities including forming farmer groups and providing coaching to ensure biosecurity through village-based disinfectants by involving all levels of animal health stakeholders [37].

### Prohibition of antibiotic growth promoters

Before the ban on AGPs, officially implemented in January 2018, the poultry industry in Indonesia relied heavily on AGPs to enhance growth rates and feed efficiency. AGPs were routinely added to feed to prevent disease and promote faster growth, and they were economically beneficial for farmers. However, this practice raised significant concerns about the development of antibiotic-resistant bacteria, which could carry severe implications for animal and human health. As resistant bacteria could be transferred to humans by consuming poultry products or through direct contact with animals, the use of AGPs undermined the effectiveness of antibiotics in treating human infections [38].

Following the decision to ban AGPs, the transition period was marked by significant challenges. Farmers had to quickly adapt to new regulations while looking for alternative methods to maintain productivity and prevent disease. This period saw a notable decline in growth rates and feed efficiency, as many farms struggled to achieve the same level of performance without AGPs. Moreover, the industry faced increased costs due to the need for more comprehensive biosecurity measures, vaccination programs, and alternative growth promoters such as probiotics, prebiotics, and organic acids. In addition, the transition required substantial investments in infrastructure and farmer education, increasing the financial burden on poultry producers.

Following the AGP ban, the Indonesian poultry industry began to stabilize and adapt to the new regulatory landscape. The use of alternative growth promoters became widespread, and improvements in biosecurity practices were evident at many farms. The AGP alternatives researched and employed in Indonesia are presented in Table 7. The poultry industry has shifted toward using natural growth promoters such as probiotics, prebiotics, phytobiotics, and organic acids to maintain gut health and improve feed efficiency. Organic acids have also been used to reduce gut pH and inhibit pathogenic bacteria, contributing to better overall flock health. Probiotics such as *Lactobacillus* and *Bacillus* have effectively improved digestion and nutrient absorption [39]. Prebiotics support the growth of beneficial gut bacteria, whereas phytobiotics and organic acids act as natural antimicrobials and growth enhancers. These alternatives, while initially costly and challenging to implement, have gradually proved effective in maintaining productivity levels.

### Avian influenza and Newcastle disease virus outbreak

Avian influenza, Newcastle disease, and coccidiosis develop rapidly under low biosecurity conditions and pose a significant risk to the poultry population and farming productivity. The increasing prevalence of these diseases requires strict implementation of biosecurity measures and possibly greater use of veterinary drugs, which would further increase production costs. Indeed, the HPAI H5N1 virus has remained endemic in Indonesian poultry since 2003, causing significant economic losses to commercial and small-scale poultry operations. In areas with high infection rates, the virus has been found in 32 of 34 provinces, leading to the death of millions of birds and the closure of many farms [40].

**Figure 9:**
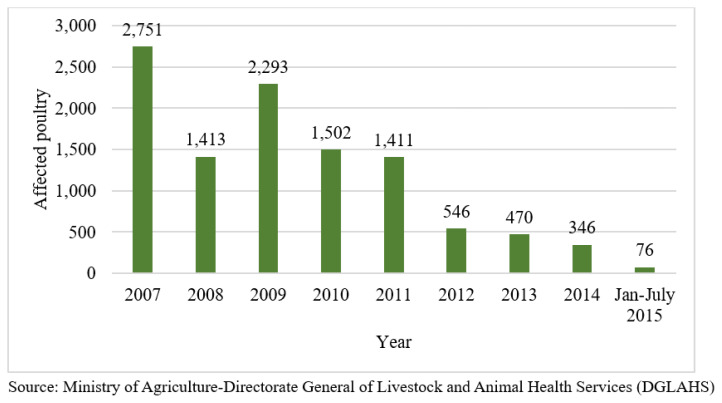
Distribution of avian influenza cases in poultry in Indonesia (affected poultry). Data from Food and Agriculture Organization of the United Nation [57].

According to the standard naming convention for the hemagglutinin (HA) gene of the HPAI H5N1 virus, HA evolved from clade 2.1 into several subclades as the virus circulated among poultry in Indonesia from 2003 to 2010 [41]. Since the detection of the first highly pathogenic avian influenza virus in Indonesia in 2003, the disease has caused the death of millions of birds and disrupted the livelihoods of many people who depend on poultry-keeping. Notably, in 2015, there was a significant decline in avian influenza cases in Indonesia, with only 76 cases (Figure 9). The number of avian influenza virus cases has continued to decline, and most recently, cases have been reported in only three provinces.

**Figure 10:**
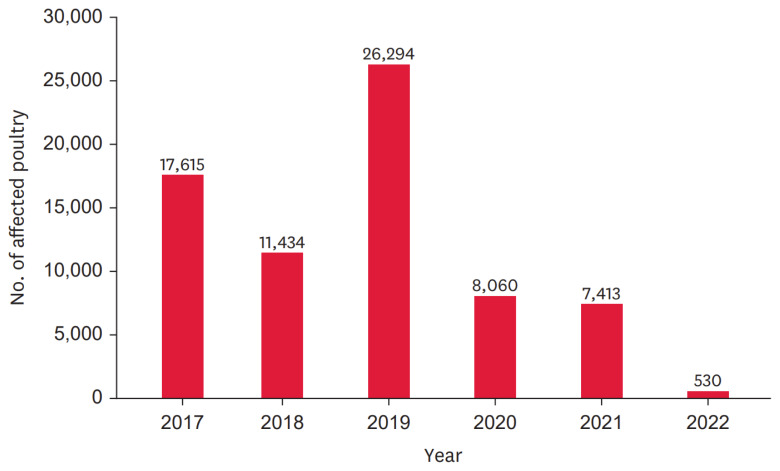
Distribution of Newcastle disease in affected poultry in Indonesia, 2017–2022. Adapted from Dharmayanti et al [43] with permission of CC-BY-NC.

The first reported outbreak of Newcastle disease occurred on Java Island in 1926. To this day, disease remains endemic to Indonesia, with ongoing outbreaks affecting free-range and commercial poultry farms [42]. According to data from the Indonesian Ministry of Agriculture [43], Newcastle disease continues to affect poultry populations across various provinces in Indonesia. There were 8,060 cases of Newcastle disease reported in 2020, but this number declined in 2021 and 2022 to 7,413 and 530 infected birds, respectively (Figure 10), which decreased by 92.85%.

The implementation of strict biosecurity measures and mandatory vaccines can be a solution for preventing diseases, especially avian influenza and Newcastle disease. Several factors should be taken when selecting the most effective and practical vaccination program, such as required level of protection, the immune status of the poultry, the pathogenicity strains, correlation with other poultry diseases, the method of vaccine application, and monitoring the immune response [43]. Newcastle disease and avian influenza can occur simultaneously in poultry, causing significant losses for poultry farmers. Chicken vaccination can be carried out using live vaccines, inactivated vaccines, or a combination of both. Vaccine manufacturers have developed combination vaccines as an effort to control newcastle disease and avian influenza in Indonesia [44]. Research from Kencana et al [44], stated that the combination vaccine also has several advantages, including the ability to be given at the same time to birds, thereby reducing the stress levels that may arise after vaccination. The cost of poultry farming can also be reduced by using the inactivated newcastle disease-avian influenza combination vaccine. In broiler chickens, a single vaccination with the inactivated vaccine is sufficient, whereas in layer chickens, revaccination is required before the laying period to trigger a protective secondary immune response, safeguarding the chickens against newcastle disease amd avian influenza cases in the field.

## CONCLUSION

Poultry farming in Indonesia plays a crucial role in providing affordable animal protein sources for the community; however, its sustainability faces several challenges. Fluctuations in feed prices, which continue to rely heavily on imports, the impact of climate change, and regulations related to antibiotic use are the main issues affecting the sector’s efficiency and productivity. The current situation demonstrates that the poultry industry is still struggling to balance supply and demand, especially with limited access to technology and wider markets for smallholder farmers. Therefore, achieving sustainability for poultry farming in Indonesia, collaborations among the government, academia, and the private sector are needed. Moreover, applying appropriate technology, improving regulations, and increasing the capacity of farmers to manage external challenges are key for maintaining and increasing the productivity of this sector in the future.

## Figures and Tables

**Table 1:** Import of day-old-chick broiler grandparent stock (birds) in 2021–2023^1)^

Month	2021	2022	2023
January	-	-	-
February	47,120	59,930	47,000
March	68,226	48,950	39,503
April	60,780	59,303	50,485
May	81,726	59,463	94,735
June	85,250	113,748	71,882
July	24,049	41,975	81,586
August	77,604	74,134	26,391
September	58,769	48,023	111,846
October	38,591	76,059	52,173
November	69,038	57,616	59,546
December	38,592	2,217	56,136
Total	649,745	641,418	691,283

1) Data from Association of Poultry Breeding Companies [45].

**Table 2:** Import and export volumes of poultry commodities (tons) in 2020–2022^1)^

Commodity	Year		

	2020	2021	2022
Import volume			
Chicken	66.16	58.81	64.11
Chicken meat	419.47	277.67	34.56
Turkey meat	-	-	0.08
Egg	2,027.55	1,999.30	2,036.40
Export volume			
Chicken	22.32	17.48	23.60
Chicken meat	594.53	652.56	477.78
Turkey meat	26	21.17	3.23
Egg	78	52.11	51.04

1) Data from Direktorat Statistik Peternakan, Perikanan, dan Kehutanan [2].

**Table 3:** Soybean import data for Indonesia by country of origin in 2017–2023^1)^

Country of origin (kg)	2017	2018	2019	2020	2021	2022	2023
United States	2,637,125	2,520,253	2,513,311	2,238,480	2,152,633	1,928,076	1,949,365
Canada	12,103.98	54,531.27	128,911	229,644	232,009	287,991	271,280
Argentina	5,000.00	-	-	633.02	89,951.00	60,823.00	23,127.00
Brazil	500.88	-	18,900.00	0.00	9,238.30	41,735.00	24,220.00
Malaysia	9,505.47	10,413.11	8,683.48	6,363.13	5,547.50	5,208.30	6,331.70
France	-	126.81	230.95	120.74	212.40	-	40.00
India	0.00	-	-	0.00	76.50	-	5.50
Others	7,678.74	484.72	48.77	45.83	22.50	895.80	58.20
Total	765,604.82	814,363.36	750,070.72	606,730.27	722,711.90	724,746.40	724,746.40

1) Data from Direktorat Statistik Peternakan, Perikanan, dan Kehutanan [2].

**Table 4:** Food prices of protein sources in Indonesia^1)^

Comodity	Price (Indonesian Rupiah, $)^2)^			

	2021	2022	2023	2024
Beef (per kg)	106,087 ($6.59)	120,688 ($7.50)	126,398 ($7.85)	123,578 ($7.68)
Milk (per litre)	15,005 ($0.93)	13,880 ($0.86)	14,521 ($0.90)	15,058 ($0.94)
Pork (per kg)	105,179 ($6.53)	139,261 ($8.65)	109,872 ($6.83)	109,098 ($6.78)
Chicken meat (per kg)	30,690 ($1.90)	31,630 ($1.97)	32,226 ($2.00)	33,180 ($2.06)
Egg (per kg)	24,530 ($1.5^2)^	26,260 ($1.63)	26,430 ($1.64)	28,410 ($1.77)

1) Data from Ministry of Agriculture [21].

2) As per exchange rate on August 7, 2024.

**Table 5:** Data recap of feed mills In Indonesia, 2023^1)^

No.	Company group	Number of feedmills	Capacity (MT per year)	Production estimate (MT per year)
1	CPI Group	16	8,780,000	7,600,000
2	JCI Group	18	5,700,000	4,140,000
3	NHI Group	9	1,700,000	1,550,000
4	CJ Group	6	1,530,000	856,500
5	Malindo Group	7	1,380,000	1,380,000
6	De Heus Group	5	1,320,000	1,170,000
7	Others	46	7,705,200	6,217,560

1) Data from Association of Animal Feed Companies [22].

CPI, Charoen Pokphand Indonesia; JCI, Japfa Comfeed Indonesia; NHI, New Hope Indonesia; CJ, Cheil Jedang.

**Table 6:** Heat stress index (HSI)^1)^ in Indonesia, 2023

Month	West Indonesia			East Indonesia		

	Minimal heat stress index	Maximal heat stress index	Average heat stress index	Minimal heat stress index	Maximal heat stress index	Average heat stress index
January	142.00	185.84	163.08	121.20	195.00	164.40
February	142.44	184.14	163.72	127.40	193.20	166.40
March	148.36	186.76	164.44	125.20	191.20	166.40
April	141.88	182.56	163.24	114.80	193.20	163.40
May	145.78	184.64	163.60	116.60	193.20	158.80
June	146.24	187.84	163.52	109.80	187.60	155.80
July	139.08	182.12	159.34	105.20	184.60	150.00
August	139.54	177.74	155.52	101.20	186.40	151.00
September	145.36	187.20	162.80	110.80	186.20	155.40
October	135.88	180.80	155.86	107.60	192.00	159.20
November	149.96	187.10	165.42	128.40	193.80	161.20
December	134.00	185.72	161.96	118.40	195.00	163.40

1) HSI is calculated based on formula according to Palupi [48].

**Table 7:** Research about AGP alternatives in Indonesia

Natural growth promoters	Dosage	Research topic	Animal	Result	Source
a. Acid PAK b. *Probalac* L c. OGC Soluble	a. 1 L/6,000 birds b. 25 mL/1,000 birds c. 100 g/1,000 birds	Evaluation of the effect of probiotics and organic acid supplementation on drinking water	Layer chicken	Probiotics can control pathogenic bacteria and maintain the immune system while organic acids can lower the pH of the digestive tract to suppress microbial growth	Varhan [49]
*Lactococcus* and *Bacillus* spesies probiotics	10^8^ cell/g	Influence of *Lactococcus* and *Bacillus* species probiotics	Broiler chicken	The feed efficiency of broiler chickens increased	Lena et al [39]
*B. coagulans* D3372	10^5^ cfu/g	Effects of dietary B. coagulans D3372 supplemention as probiotics on broiler	Lohmann broiler chicken	Increasing body weight, performance, and income over feed and chicken cost	Zainuddin et al [50]
Curcuma xanthorrhiza and garlic	2% of feed	Effects of herbal formulation on the productivity of stunted broilers	Stunted broiler (narrative review)	Improved growth and performance of the broiler	Lestari et al [51]
Acidified turmeric or black pepper powder	1% of feed	Effect of acidified turmeric or black pepper on the growth performance and meat quality of broiler chickens	Lohmann broiler	Reduced abdominal fat content and improved meat characteristics of the broiler	Sugiharto et al [52]
Natural feed additives Curcumin Gingerol Epigoitrin Andrographis paniculata Tyramine Cianidanol Cyranoside	0.25%–1% of feed	Exploring the potential of natural feed additives from herbs as an alternative for AGPs	Mojosari layer duck	Natural feed additives as natural growth promoter did not impact enzyme performance, regulate insulin levels in ducks, enhancing egg quality and production	Djunaidi et al [53]
Binahong (*Anredera cordifolia*) leaf extract	100–250 mg/kg body weight	Effect of Anredera cordifolia extract in water on the performance of the broiler	Broiler strain MB 202	The extract had no impact on growth performance	Hasiib et al [54]
Blends of *Zingiber officinale, Curcuma domestica, Kaempferia galanga, Curcuma xanthorrhiza and probiotic Lactobacillus acidophilus, Saccharomyces cerevisiae, Lactobacillus bulgaricus, Bacillus subtilis, Bacillus megaturium, Lactobacillus plantharum* and *Lactobacilus sullivarius*	1.5 mL/L of water	Effect of the probiotic and herb mix on the performance, carcass, and physical quality of broilers	Broiler	Improved feed conversion ratio and carcass traits of broilers	Sukmaningsih and Rahardjo [55]
*Phaleria macrocarpa leaf extract*	More than 20% of drinking water	Supplementation with Phaleria macrocarpa leaf extract significantly reduces E. coli infection in broiler	Broiler	Reduced E. coli counts in the intestine of broilers	Diyantoro and Rochmi [56]

AGP, antibiotic growth promoter; *B. coagulans*, *Bacillus coagulans*; *E. coli*, *Escherichia coli*.
